# MicroRNA-Mediated Regulation of HMGB1 in Human Hepatocellular Carcinoma

**DOI:** 10.1155/2018/2754941

**Published:** 2018-01-31

**Authors:** Jianing Yan, Shibo Ying, Xiujun Cai

**Affiliations:** ^1^Department of General Surgery, Sir Run Run Shaw Hospital Affiliated to Medical College of Zhejiang University, Hangzhou 310016, China; ^2^Institute of Occupational Diseases, Zhejiang Academy of Medical Sciences, Hangzhou 310013, China

## Abstract

High-mobility group box 1 (HMGB1) is a potential therapeutic target and novel biomarker in a variety of malignant tumors, including hepatocellular carcinoma (HCC). More recently, a number of microRNAs (miRNAs) are identified as a class of regulators for broad control of HMGB1-mediated biological actions in eukaryotic cells. In this review article we will describe representative miRNAs involved in regulating the HMGB1 signaling pathways in HCC cell lines and/or animal models. We also propose the possible mechanisms underlying the miRNA/HMGB1 axis and discuss the future clinical significance of miRNAs targeting HMGB1 molecule for HCC therapy.

## 1. Introduction

Hepatocellular carcinoma (HCC) is the sixth most common malignant tumor and the third leading cause of cancer-related death. In 2013, 792,000 new cases of HCC occurred globally, and 818,000 of these cases resulted in death [1]. Although treatment of HCC has changed over the last 10 years and even though patients are able to choose different therapies that improve survival, effective therapeutic strategies are still severely needed. Particularly, the identification of novel biomarkers and molecular targets would provide HCC patients with better classification schemes and treatment options [2].

MicroRNAs (miRNAs) are tiny noncoding RNAs that are 20–22 nucleotides in length. The functions of miRNAs are associated with several crucial biological processes, such as cell proliferation, differentiation, and apoptosis [3]. The 5′ end of the miRNA (“seed” region) targets the coding and 3′ untranslated regions (3′ UTRs) of the messenger RNA (mRNA) through specific paired interactions. Thus, miRNAs regulate the assembly of the miRNA-containing ribonucleoprotein (miRNP) complex and the Argonaute/EIF2C (AGO) proteins, which eventually leads to mRNA degradation [3]. Dysregulation of miRNAs can drive tumorigenesis, and, thus, identification of miRNAs as serum biomarkers may serve as a useful diagnostic approach to cancer [4]. Several studies have reported the use of miRNAs as biomarkers and their impact on the development of therapeutic strategies in breast cancer [5], colorectal cancer [6], cervical cancer [7], lung cancer [8], thyroid cancer [9], soft tissue sarcoma [10], prostate cancer [11], and pancreatic cancer [12]. The roles of miRNAs in HCC especially are being heavily studied [13]. For instance, Tsang et al. have reported that miR-125b functions as a tumor suppressor by inhibiting eukaryotic translation initiation factor 5A2 (eIF5A2) in HCC cell lines, indicating the negative regulatory impact of miR-125b on HCC [14]. Wang et al. have suggested that overexpression of miR-215 in HCC cells is correlated with upregulated chemoresistance, via targeting of dihydrofolate reductase (DHFR) and thymidylate synthase, and proliferation, via effects on P21 expression [15].

The high-mobility group box 1 protein (HMGB1) is a chromatin-binding factor that targets DNA and facilitates the process of transcriptional protein assembly. First discovered in 1973, HMGB1 is presumably released from dead cells and secreted by inflammatory cells, and HMGB1 plays central roles in various disease states [16], including autoimmune disease [17], ischemia reperfusion injury [18], inflammation, and cancer [19, 20]. By acting as a damage-associated molecular pattern (DAMP), HMGB1 binds with high affinity to several receptors, such as the receptor for advanced glycation end products (RAGE) and Toll-like receptor- (TLR-) 2, TLR-4, and TLR-9, mediating the immune response to necrosis and immune cell invasion to trauma, pathogens, and sepsis [21]. Additionally, overexpression of HMGB1 is associated with several cancer characteristics. Binding to RAGE, HMGB1 enhances cell migration and tumor metastasis, thereby promoting cancer development [22, 23]. Several studies have focused on the association between HMGB1 expression and HCC. Serum HMGB1 can be used as a marker to evaluate tumor stage and predict HCC prognosis in patients [24, 25]. Additionally, HMGB1 regulates cell proliferation [26], cell differentiation [27], and tumor metastasis via various signaling pathways [28–30] in HCC. Since HMGB1 has potential to function as a diagnostic biomarker or curative target, the function of HMGB1 in HCC has been a hot research topic in recent years.

## 2. Crucial Roles of HMGB1 in HCC

The roles of HMGB1 probably are of crucial importance in HCC tumorigenesis and tumor development. In fact, HCC is usually characterized as a typical inflammation-related carcinoma. Proinflammatory mediator HMGB1 is currently believed to be secreted actively by hepatitis viruses-infected hepatocytes in the chronic inflammatory status of the liver [31]. There is increasing evidence that HMGB1 is involved in the process of HCC formation and progression, such as cell proliferation, differentiation, angiogenesis, metastasis, inflammation, and immune function in* in vitro* and* in vivo* HCC models [31, 32]. The interaction of extracellular HMGB1 with its receptors RAGE/TLRs on cell surface may activate intracellular NF-*κ*B pathways, inducing the elevation of leukocyte adhesion molecules and proinflammatory cytokines, thereby promoting inflammation and creating a sustained tumor microenvironment, and ultimately resulting in HCC [33]. It was also reported that HMGB1 promotes the growth of HCC cell line* in vitro*. Inhibition of HMGB1 by RNA interference not only represses the ability of cell proliferation, migration, and invasion, but also enhances apoptosis through NF-*κ*B pathways [34]. Furthermore, previous studies indicated a close correlation between p53 and HMGB1 which plays an important role in HCC tumorigenesis. Of note, p53 was originally considered to be a tumor suppressor. However, it was demonstrated that sustained p53 activation could accelerate protumorigenic liver inflammation in rat liver by inducing release of HMGB1 [35]. More interestingly, HMGB1 and p53 also form a complex which regulates the balance between tumor cell death and survival [36]. In addition, the effect of HMGB1 is also thought to be associated with metastasis of HCC. HMGB1 activates caspase-1 through RAGE/TLR4 signaling pathway in hypoxic HCC cells, leading to cleavage and release of proinflammatory cytokines such as IL-1*β* and IL-18, which in turn promotes the invasiveness and metastases of HCC [28]. Altogether, these findings suggest that HMGB1 is a powerful carcinogenic mediator and serves as potential therapeutic target for HCC.

## 3. MicroRNAs Regulate HMGB1 Signaling Pathways

The crucial roles of HMGB1 signaling in tumor development and the progression of various processes are now well established. However, the mechanisms and extent to which miRNAs contribute to alterations in HMGB1 expression and/or activities in pathological conditions remain poorly understood in HCC. Representative miRNAs and their effects on HMGB1 in HCC are outlined in Table 1. In this section, we describe the main findings from relevant studies regarding miRNAs, which regulate the HMGB1 signaling pathways. These findings may provide a better understanding of the biological mechanisms and potential clinical significance of miRNAs in HCC.

### 3.1. miR-200a

miR-200a belongs to the miRNA-200 (miR-200) family, which contains five members that compose two clusters [42]. Cluster I miR-200s, which include miR-200b, miR-200a, and miR-429, are located in chromosome 1 [43]. As a member of the miR-200 family, miR-200a influences the epithelial-to-mesenchymal transition (EMT) and metastasis of various tumor types [44]. A negative correlation reportedly exists between the miR-200a level and tumorigenesis, angiogenesis, and metastasis through cell signaling pathways in breast cancer [45, 46], ovarian cancer [47, 48], endometrial cancer [49], meningioma [50], pituitary cancer [51], renal cancer [52], prostate cancer [53], oral cancer [54], nasopharyngeal carcinoma [55], and HCC [37].

miR-200a may regulate the neoplastic transition of the hepatic oval cell by directly targeting the *β*-catenin (CTNNB1) pathway [56]. A recent study has described the role of miR-200a in regulating HCC cell growth via direct targeting of HMGB1 [37], and the authors have reported the interaction between long noncoding RNA (lncRNA) TP73-AS1 and miR-200a during the process of HCC cell proliferation [37]. According to their findings, miR-200a inhibits HMGB1 expression. Additionally, high lncRNA-TP73-AS1 expression in patients is correlated with a worse prognosis. The protein levels of HMGB1, RAGE, and NF-*κ*B are significantly decreased after TP73-AS1 knockdown. More detailed studies have shown that TP73-AS1 competes with HMGB1 for miR-200a binding. The interaction between miR-200a and TP73-AS1 is consequently regulated by HMGB1/RAGE expression and downstream NF-*κ*B targets, such as inflammatory cytokines. Reduced binding between TP73-AS1 and miR-200a promotes the inhibitory actions of miR-200a toward HMGB1 and ultimately delays HCC progression [37].

### 3.2. miR-320a

miR-320a is downregulated in various malignant diseases. One study has demonstrated that miR-320a represses proliferation and metastasis and enhances irradiation-induced apoptosis by directly targeting STAT3 signals in lung adenocarcinoma cells [57]. Several researchers had reported that, by targeting *β*-catenin, miR-320 suppresses the proliferation of colon cancer cells [58] and has potential as a prognostic biomarker for colorectal cancer [59]. Other studies have also suggested that miR-320a can inhibit tumor growth in breast cancer [60], glioblastoma [61], gastric cancer [62], and acute lymphoblastic leukemia [63].

The roles of miR-320a in HCC are also under discussion. Yao et al. have reported that miR-320a deregulates guanine nucleotide-binding protein G(i) *α*-1 subunit (GNAI1) and facilitates the migration and invasion of HCC [64]. Yuan et al. have reported that miR-320a has a suppressive effect on the stemness features of HCC cells by repressing CTNNB1 signals and that association of lncRNA-DANCR with CTNNB1 decreases the inhibitory actions of miR-320a on CTNNB1 [65].

In an investigation of the interaction between miR-320a and HMGB1, one research group demonstrated a remarkable decline in miR-320a in HCC patients [38]. Because HMGB1 is a known direct target of miR-320a, the expression level of miR-320a might regulate the invasion and metastasis of HCC cells by targeting the HMGB1 pathway, partly indicating the potential antimetastasis role of miR-320a in HCC [38].

### 3.3. miR-505

Several studies discuss the role of miR-505 in different cancers. miR-505 is reportedly associated with apoptosis, and studies of drug-resistant human breast cancer cell lines have shown that miR-505 inhibits proliferation by inducing apoptosis [66]. However, high expression of miR-505 is strongly associated with a poor prognosis in lymph node-negative breast cancer patients [67]. Additionally, several reports have indicated that miR-505 plays a suppressor role in cervical carcinoma by targeting the Frizzled-4 gene (FZD4) [68] and in endometrial cancer by targeting transforming growth factor-*α* (TGF-*α*) [69]. miR-505 expression is correlated with the potential of establishing a biomarker of the imatinib therapeutic response in chronic myeloid leukemia patients [70]. Interestingly, Qin et al. have reported that miR-505 is upregulated in a nasopharyngeal cell line but that miR-505 surprisingly still inhibits metastasis and suppresses the expression of EMT markers, which are the genes associated with cell migration and invasion [71].

Similarly, the results of studies in HCC have also revealed several latent functions for miR-505. Dysregulation of miR-505 might be associated with HCC [72]. A recent article has presented several studies showing that miR-505 is decreased in various HCC cell lines. miR-505 attenuates HCC cell proliferation and invasion by inhibiting HMGB1, suggesting potential antitumor roles for miR-505 in HCC [39].

### 3.4. miR-129-2

miR-129-2 belongs to the miR-129 family, which presumably functions negatively in different cancers. miR-129-2 suppresses proliferation, migration, and invasion of renal carcinoma [73], esophageal carcinoma [74], breast cancer [75, 76], and lung cancer [77]. Furthermore, the miR-129-2 gene is reportedly hypermethylated in endometrial cancer [78], gastric carcinoma [79], osteosarcoma [80], and glioma [81]. miR-129-2 downregulates the expression of SOX4 [74], an oncogene of the SRY-related HMGB family, and epigenetic deregulation of miR-129-2 results in overexpression of SOX4 in endometrial cancer [78] and gastric carcinoma [79]. Lu et al. have found frequent DNA methylation of miR-129-2 in HCC and have suggested the potential clinical utility of miR-129-2 as a diagnostic biomarker for HCC [82]. Chen et al. have reported that methylation-mediated repression of miR-129-2 might promote SOX4 expression and HCC progression [83].

The relationship between miR-129-2 and HMGB1 is also under exploration. Yang et al. have reported that the expression of miR-129-2 in glioma cells is regulated by DNA methylation and that miR-129-2 inhibits glioma cell growth and promotes apoptosis by directly targeting HMGB1 [81]. Another group from China have reported that miR-129-2 is remarkably declined in HCC cells [40]. The miR-129-2 level is correlated with venous infiltration, a high Edmondson-Steiner grade, and an advanced tumor-node-metastasis (TNM) stage and serves as an independent prognostic factor to indicate overall survival and disease-free survival. Further investigations have indicated that miR-129-2 suppresses AKT phosphorylation and downregulates matrix metalloproteinase 2/9 (MMP2/9) expression. Suppression of p-AKT significantly represses HMGB1-mediated HCC cell migration and invasion. Additionally, miR-129-2 is regulated by DNA methylation. Demethylation of miR-129-2 has an inhibitory impact on cell growth, partly though inhibition [40].

### 3.5. miR-325

A few studies have reported functions for miR-325 in cancer. Wong et al. have shown that miR-325 is upregulated in squamous cell carcinoma of the tongue [84]. Yao et al. have reported that in non-small-cell lung cancer, HMGB1 is negatively regulated by miR-325 and that an aberrant expression level of miR-325 affects cell invasion and proliferation [85]. One study that investigated the expression of miR-325 and its impact via HMGB1 targeting in HCC has shown that the expression level of miR-325 is negatively correlated with HMGB1 in HCC. miR-325 functions as a tumor suppressor by targeting HMGB1 and might serve as a potential prognostic marker for HCC [41].

### 3.6. miR-21

In contrast to the miRNAs described above, miR-21 is frequently overexpressed in different malignant tumor cell lines and tissues, such as glioblastoma [86], breast cancer [87, 88], myeloma [89], colon cancer [90], prostate cancer [91], B-cell lymphoma [92], and lung cancer [93]. Further functional studies have indicated that miR-21 plays a key role in tumor progression through its association with antiapoptotic effects, radical invasion, and metastasis [86, 88]. miR-21 is distinguished from the other miRNAs mentioned in this review because miR-21 has been described as an oncomiR due to its facilitative impact on tumorigenesis [94].

The link between miR-21 and HCC has also has been thoroughly studied. Wang et al. have reported that the serum miR-21 level is associated with HCC prognosis and can even be an earlier and more precise biomarker than AFP [95]. Huang et al. have indicated that elevated expression of miR-21 is associated with HCC progression [96]. Guo et al. have shown that, combined with AFP, circulating miR-21 might enable an HCC diagnosis, particularly in AFP-negative patients [97]. Additionally, Hu et al. have shown that lncRNA growth arrest-specific transcript 5 (GAS5) moderates the expression of miR-21 and ultimately attenuates the migration and invasion of HCC cells [98]. Different mechanisms or pathways of miR-21 that promote the malignancy of HCC have also been identified. By using locked nucleic acids, Najafi et al. have generated a model of miR-21 degradation in an HCC cell line to assess the effect of miR-21 degradation on cell viability and apoptosis and ultimately have found that downregulation of miR-21 decreases HCC cell viability by inducing apoptosis and necrosis [99]. Jiang et al. have reported that the liver cancer stem cells are enhanced by overexpression of miR-21, which then leads to high invasion and migration of HCC [100]. Li et al. have confirmed that miR-21 and miR-183 negatively regulate cytokine signaling 6 (SOCS6), a negative regulator of the cytokine receptor pathway, and subsequently modulate tumor growth and invasion [101]. Moreover, several studies have revealed the connection between hepatitis B virus (HBV) X protein (HBx), miR-21, and HCC. Qiu et al. have indicated that HBx downregulates the expression of programmed cell death 4 (PDCD4), whose expression level is low in HCC patients and which can attenuate carcinogenesis, partially via miR-21 [102]. Li et al. have observed that upregulation of miR-21 is induced by the HBx-interleukin-6- (IL-6-) STAT3 pathway and that miR-21 is a critical factor in the process of HBx-induced transformation, suggesting the promoting effect of miR-21 in early carcinogenesis [103].

One research group from China has focused on the link between liver inflammation and HCC under the effects of HMGB1 and dysregulation of miR-21 [30]. The researchers have identified a balanced level of expression between HMGB1 and miR-21 in HCC cells and patients and have verified that these levels are regulated by the IL-6-STAT3 signaling axis. Further studies have shown that, through the IL-6-STAT3 pathway, HMGB1 signaling elevates the level of miR-21 expression and subsequently inhibits posttranscriptional processing of the MMP inhibitors RECK and TIMP3. By attenuating RECK and TIMP3 which could repress the malignant characteristics of HCC, miR-21 enhances the activity of MMPs and ultimately promotes HCC progression [30].

## 4. Biological Mechanisms of the MicroRNA/HMGB1 Axis

**Figure 1:**
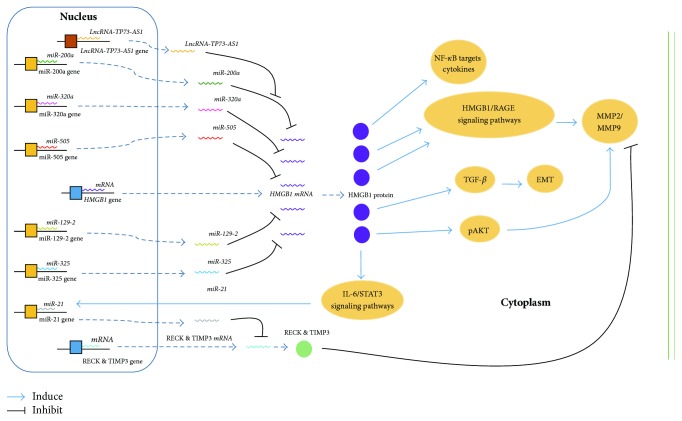
Schematic representation of microRNAs regulating HMGB1-mediated mechanisms.

HCC releases DAMP proteins, such as HMGB1, which interact with their receptors, triggering a proinflammatory signaling cascade that contributes to tumor progression [104, 105]. HMGB1 can be recognized by siRNA and activate downstream signaling molecules. Although the linkages between miRNAs and HMGB1 in HCC have been discussed thoroughly above, a universal conclusion of the molecular mechanisms of the interactions between miRNAs and HMGB1 is required. Based on miRNAs described in Section 3, we propose a hypothesis that the mechanism of biological mechanisms of the miRNA/HMGB1 axis as shown in Figure 1.

To our knowledge, gene expression in eukaryotic cells is regulated at multiple levels, including transcriptionally, posttranscriptionally, translationally, posttranslationally, and epigenetically. Furthermore, miRNAs mainly participate in regulating progression at the posttranscriptional level. Similar to the biological behaviors of these tiny noncoding RNAs, several specific miRNAs bind to the HMGB1 mRNA, via the particular targeting effect of the “seed” region of miRNAs in the coding region and 3′ UTR of the mRNA, thus guiding the process of HMGB1 mRNA degradation, influencing downstream signal pathways, such as HMGB1-RAGE-MMP2/MMP9 signaling [40], and ultimately altering the malignant tumor characteristics of HCC (Figure 1). Additionally, several other molecules take part in the miRNA-HMGB1 axis. Multiple lncRNAs [37], while competing with HMGB1 mRNAs, interact with several peculiar miRNAs, which explains the miRNA posttranscriptional regulation of HMGB1. Furthermore, miRNAs may also regulate the production of downstream molecules. The HMGB1 protein stimulates HCC cells to generate oncomiRs, which inhibit tumor suppressors such as RECK and TIMP3 [30], and promote HCC malignancy.

Altogether, the miRNA-HMGB1 axis probably is an important mechanism that is active during the posttranscriptional regulatory processes that target HMGB1 in HCC.

## 5. Future Perspective Regarding Clinical Significance

Several recent studies about the linkages between miRNAs and HMGB1 have highlighted the availability of miRNAs that can serve as prognostic predictors of HCC in patients. For example, Liu et al. have noted that the relationship between the miR-129-2 level and advanced malignant features is reversely correlated, which might provide hints toward patient prognosis [40]. Additionally, Li et al. have shown that the negative correlation between miR-325 and tumor size, TNM stage, and metastasis in HCC highlights miR-325 as an independent prognostic factor for HCC patients [41]. Therefore, these findings suggest that the miRNA expression profile will serve as a credible tool for diagnostics, tumor staging, and prognostic predictions of HCC in the future.

Moreover, some studies, which have confirmed the functions of miRNAs on HMGB1 in different signaling pathways during the regulation of tumor progression, have suggested several novel available therapies that are based on the miRNA-HMGB1 axis to combat HCC. Overexpression of miRNAs that target HMGB1 mRNA or silence oncomiRs that inhibit tumor suppressor genes might reduce the production of HMGB1, block downstream signaling pathways, and consequently downregulate HCC malignancy. Combined with current methods of treatment, including surgery, chemotherapy, radiotherapy, radiofrequency ablation, hepatic arterial chemoembolization (TACE), and immunotherapy, the regulatory effects of miRNA-HMGB1 in HCC may play important roles in targeted therapy and precision medicine in the future.

## Figures and Tables

**Table 1:** List of microRNAs involved in HMGB1 signaling pathways.

miRNA	Binding site	Biological effects on HMGB1	Biological effects on phenotype	Possible involved signaling pathway	Clinical significance	Cell lines	Animal model	Ref
miR-200a	lncRNA-TP73-AS1 3′UTR of HMGB1 mRNA (position 2724–2730)	miR-200a negatively regulates HMGB1 expression. TP73-AS1 competes with HMGB1 for miR-200a binding.	Inhibition of cell proliferation	HMGB1/RAGE and NF-кB targets cytokines	High lncRNA-TP73- AS1 expression in HCC is correlated with poorer prognosis.	HCCLM3, MHCC97L, SMMC772, Hep3B, HepG2	-	[37]

miR-320a	3′UTR of HMGB1 mRNA	miR-320a suppresses the expression of HMGB1.	Suppressing the invasion and metastasis of cells	HMGB1-RAGE-MMP2/MMP9 signaling	The decrease of miR-320a is associated with the invasion and metastasis.	HepG2, SK-hep-1	-	[38]

miR-505	3′UTR of HMGB1 mRNA (positions 417–424)	miR-505 negatively regulates HMGB1 expression in cells.	Inhibition of cell proliferation, invasion, and EMT	TGF-*β*-induced EMT	-	QGY7703, SMMC7721, MHCC97	-	[39]

miR-129-2	3′UTR of HMGB1 mRNA (positions 387–394)	HMGB1 is a downstream mediator of the biological function of miR-129-2.	Suppressing HCC migration and invasion	HMGB1-pAKT-MMP2 /MMP9 signaling	miR-129-2 is inversely correlated with venous infiltration, high Edmondson-Steiner grading, and advanced TNM stage. An independent prognostic factor for OS/DFS.	HepB3, Huh7	BALB/c nude mice	[40]

miR-325	3′UTR of HMGB1 mRNA	miR-325 negatively regulates HMGB1 expression.	Inhibition of cell invasion and proliferation	-	miR-325 is highly associated with HCC tumor size, TNM stage, and metastasis of patients. An independent prognostic factor for OS.	SMMC-7721, Hep3B, HepG2, Huh7, Bel7404	-	[41]

miR-21	3′-UTRs of RECK and TIMP3 mRNA	HMGB1 signaling increases miR-21 expression to mediate the enhanced activity of MMPs through RECK/TIMP3.	Promoting cell invasion, migration, and proliferation	HMGB1-IL-6/Stat3 signalingRECK/TIMP3-MMP	-	Primary HCC cells, Huh7, HepG2, HepB3	Athymic nude mice	[30]

3′UTR: 3′ untranslated region; RAGE: receptor for advanced glycation end-products; MMP: matrix metalloproteinases; EMT: epithelial-mesenchymal transition; TNM: tumor-node-metastasis; OS: overall survival; DFS: disease-free survival; RECK: reversion-inducing cysteine-rich protein with Kazal motifs; TIMP3: metalloproteinase inhibitor 3. Ref: references.
